# Supplementary material for: Strategies for recovery of imbalanced full-scale biogas reactor feeding with palm oil mill effluent
Source: PeerJ. 2021 Jan 7;9:e10592. doi: 10.7717/peerj.10592 (PMC7797170; doi:10.7717/peerj.10592)
Supplement: Supplemental Information 3 [file peerj-09-10592-s003.docx]

| Band | Organism | %Identity | Best match in GenBank  (Accession Number) |
| --- | --- | --- | --- |
| 1 | *Desulfotomaculum* sp. | 100 | NR_044832.2 |
| 2 | *Blautia* sp. | 84 | NR_112789.1 |
| 3 | *Clostridium* sp. | 78 | NR_115345.1 |
| 4 | *Anaerostipes* sp. | 83 | NR_117139.2 |
| 5 | *Lactobacillus* sp. | 98 | JQ805621.2 |
| 6 | *Lysinibacillus* sp. | 91 | NR_146821.1 |
| 7 | *Staphylococcus* sp. | 100 | NR_113351.1 |
| 8 | *Kurthia gibsonii* | 99 | NR_119002.1 |
| 9 | *Exiguobacterium* sp. | 95 | NR_075006.1 |
| 10 | *Bacillus* sp. | 83 | NR_144712.1 |
| 11 | *Bacteroides* sp. | 96 | NR_113069.1 |
| 12 | *Selenomonas* sp. | 86 | NR_117597.1 |

**Table S1.** Molecular identification of band of bacteria during recovery by self-recovery, dilution with BE 8:2, 0.14% w/v NaOH, 0.50% w/v Ca(OH)_2_, and 8.0% w/v palm oil ash from DGGE analysis.
