# Supplementary material for: Strategies for recovery of imbalanced full-scale biogas reactor feeding with palm oil mill effluent
Source: PeerJ. 2021 Jan 7;9:e10592. doi: 10.7717/peerj.10592 (PMC7797170; doi:10.7717/peerj.10592)
Supplement: Supplemental Information 4 [file peerj-09-10592-s004.docx]

**Table S2.** Molecular identification of band of archaea during recovery by self-recovery, dilution with BE 8:2, 0.14% w/v NaOH, 0.50% w/v Ca(OH)_2_ and 8.0% w/v palm oil ash from DGGE analysis.

| Band | Organism | %Identity | Best match in GenBank  (Accession Number) |
| --- | --- | --- | --- |
| 1 | *Methanococcoides* sp. | 94 | NR_029122.1 |
| 2 | *Methanosaeta* sp. | 99 | NR_102903.1 |
| 3 | *Methanosaeta* sp. | 99 | NR_102903.1 |
| 4 | *Methanosarcina* sp. | 92 | NR_109423.1 |
| 5 | *Methanosarcina* sp. | 90 | NR_074110.1 |
| 6 | *Methanosaeta* sp. | 99 | NR_102903.1 |
| 7 | *Methanosaeta* sp. | 99 | NR_102903.1 |
| 8 | *Methanosarcina* sp. | 97 | NR_118371.1 |
| 9 | *Methanosarcina* sp. | 94 | NR_074110.1 |
| 10 | *Methanosarcina* sp. | 98 | NR_074110.1 |
